# Clozapine-Associated Myocarditis Presenting With Acute Heart Failure: Inflammation Before Injury

**DOI:** 10.7759/cureus.105736

**Published:** 2026-03-23

**Authors:** Pahresah Roomiany, Anisa Eshraghi, Faye Farber

**Affiliations:** 1 Hospital Medicine, Duke University Health System, Durham, USA; 2 Family and Community Medicine, Duke University Health System, Durham, USA

**Keywords:** antipsychotic complications, clozapine, c-reactive protein, drug induced cardiomyopathy, drug toxicity, heart failure, myocarditis

## Abstract

Clozapine is the most effective antipsychotic medication for treatment-resistant schizophrenia but is associated with rare and potentially life-threatening myocarditis. Early clinical manifestations are often nonspecific and may delay diagnosis. We report a man in his 30s who developed fever and palpitations three weeks after initiation of clozapine therapy. Laboratory testing demonstrated a marked elevation in C-reactive protein that preceded the development of cardiac biomarker elevation and new left ventricular systolic dysfunction. Transthoracic echocardiography demonstrated an ejection fraction of 30% to 35%, consistent with acute heart failure with reduced ejection fraction. Clozapine was discontinued, and guideline-directed medical therapy was initiated, resulting in complete recovery of cardiac function on follow-up imaging. This case highlights the potential role of inflammatory markers as an early signal of evolving clozapine-associated myocarditis and emphasizes the importance of early recognition and prompt drug discontinuation.

## Introduction

Clozapine is widely recognized as one of the most effective antipsychotics for treatment-resistant schizophrenia but remains underutilized due to concerns regarding serious adverse effects [1]. Although hematologic toxicity such as agranulocytosis is well known, cardiovascular complications, including myocarditis and cardiomyopathy, are less frequently recognized [2]. Clozapine-associated myocarditis most commonly occurs within the first several weeks of therapy and may progress rapidly to heart failure or arrhythmia if not recognized early [3].

Clinical presentation is often nonspecific and may resemble viral illness with fever, malaise, and sinus tachycardia preceding overt cardiac dysfunction [3]. Emerging evidence suggests that inflammatory activation may precede myocardial injury in some patients, raising the possibility that inflammatory markers may provide an early signal prompting further cardiovascular evaluation [4]. The leading hypothesis involves an IgE-mediated type I hypersensitivity reaction characterized by eosinophilic infiltration of the myocardium and subsequent inflammatory injury. Additional mechanisms include direct cardiotoxic effects of clozapine metabolites, catecholamine excess due to norepinephrine transporter inhibition, oxidative stress, and cytokine-mediated myocardial damage, all of which can impair myocardial contractility and lead to acute heart failure [5]. We present a case of clozapine-associated myocarditis in which a marked elevation in C-reactive protein preceded cardiac biomarker elevation and the development of systolic dysfunction.

## Case presentation

A man in his 30s with schizoaffective disorder, obsessive compulsive disorder, and chronic kidney disease stage IIIA secondary to prior lithium toxicity was transferred from a psychiatric facility for evaluation of several days of fever, cough, and palpitations. He denied chest pain, dyspnea, orthopnea, or lower extremity edema and had no prior history of coronary artery disease or heart failure.

Clozapine therapy had been initiated three weeks earlier for refractory psychosis. Additional medications included amlodipine, metoprolol, and losartan for treatment of hypertension. Ten days after clozapine initiation, outpatient laboratory testing demonstrated normal inflammatory and cardiac biomarkers, including C-reactive protein 1.9 milligrams per liter, high-sensitivity troponin I less than 2.7 nanograms per liter, and B-type natriuretic peptide less than 15 picograms per milliliter.

Approximately two weeks after starting clozapine, the patient developed fever and palpitations. Respiratory viral testing, including SARS-CoV-2, influenza A, influenza B, and respiratory syncytial virus, was negative.

Repeat laboratory testing obtained three days prior to admission demonstrated persistent normal troponin and B-type natriuretic peptide levels, but markedly elevated C-reactive protein at 84.5 milligrams per liter. The white blood cell count was normal, with mild eosinophilia. Over the subsequent 48 hours, C-reactive protein increased to 174.1 milligrams per liter, accompanied by elevations in troponin at 598.1 nanograms per liter and B-type natriuretic peptide at 73 picograms per milliliter, prompting emergency department evaluation (Table 1).

**Table 1:** Biomarker and Clinical Timeline CRP: C-reactive protein; BNP: brain natriuretic peptide; mg: milligrams; L: liter; ng: nanograms; pg: picograms; mL: milliliter; EF: ejection fraction

Time From Clozapine Initiation	CRP (mg/L)	Troponin (ng/L)	BNP (pg/mL)	Clinical Status
Day 10	1.9	<2.7	<15	Asymptomatic
Day 18	84.5	Normal	Normal	Fever and palpitations
Day 20	174.1	598	73	Emergency evaluation
Day 22	-	-	-	Echo EF 30-35%
Week 6	-	-	-	EF normalized

On presentation, the patient was febrile with a temperature of 38.2 degrees Celsius, tachycardic with a heart rate between 110 and 120 beats per minute, and mildly tachypneic with preserved oxygen saturation on room air. Blood pressure was 100/70 mmHg. Electrocardiography demonstrated sinus tachycardia without ischemic changes. Serum creatinine remained at baseline.

Transthoracic echocardiography demonstrated new left ventricular systolic dysfunction with an ejection fraction of 30% to 35%, consistent with acute heart failure with reduced ejection fraction (Video 1). Right ventricular function and valvular anatomy were normal.

**Video 1:** Apical Four-Chamber View on Cardiac Echocardiography

Clozapine was discontinued immediately due to concern for drug-induced myocarditis. The patient was treated with intravenous diuresis using furosemide and initiated on guideline-directed medical therapy for heart failure, including beta blockade and renin angiotensin system inhibition.

Following discontinuation of clozapine, inflammatory markers and cardiac biomarkers progressively declined, and the patient’s symptoms improved. A follow-up transthoracic echocardiography performed six weeks later demonstrated complete recovery of left ventricular systolic function.

## Discussion

Clozapine-associated myocarditis is an uncommon but potentially life-threatening adverse effect that most commonly occurs during the early phase of therapy [2]. The reported incidence varies widely across studies due to differences in surveillance strategies and diagnostic criteria [3]. Identified risk factors include younger age, male sex, and rapid dose escalation.

Clinical manifestations are frequently nonspecific and may resemble viral illness with fever, malaise, and sinus tachycardia preceding the development of myocardial dysfunction [2]. This nonspecific presentation can contribute to delayed diagnosis and underrecognition.

Increasing evidence supports an inflammatory or immune-mediated mechanism underlying clozapine-associated myocarditis [4]. Several reports suggest that inflammatory markers, particularly C-reactive protein, may rise before myocardial injury becomes clinically apparent [4,6].

This case demonstrates a clinically useful diagnostic window in which inflammatory marker elevation preceded cardiac biomarker elevation and ventricular dysfunction, suggesting that early C-reactive protein monitoring may help identify evolving clozapine-associated myocarditis before overt myocardial injury develops.

Prompt discontinuation of clozapine remains the cornerstone of management when myocarditis is suspected [3]. When identified, early myocardial dysfunction is often reversible with supportive care and guideline-directed medical therapy.

## Conclusions

Clozapine-associated myocarditis is a rare but serious complication that typically occurs during the early weeks of therapy. Because initial symptoms may be nonspecific, diagnosis can be delayed.

This case presentation is novel in its identification of inflammatory marker elevation as a potential early signal that may allow clinicians to identify evolving clozapine-associated myocarditis before the development of significant myocardial injury. Early recognition and prompt discontinuation of clozapine remain critical to preventing progression to severe heart failure, arrhythmia, or death.
